# Association between craniosynostosis and phospholipid metabolism: Insights from single-cell and transcriptomic analysis

**DOI:** 10.1097/MD.0000000000048030

**Published:** 2026-03-27

**Authors:** Kaisai Tuerxun, Junhua Wang, Zhimin Huang, Yuqi Zhang

**Affiliations:** a Tsinghua Medicine, Tsinghua University, Beijing, P. R. China; b Department of Neurosurgery, Tsinghua University Yuquan Hospital (Tsinghua University Hospital of Integrated Traditional Chinese and Western Medicine), Beijing, P. R. China.

**Keywords:** craniosynostosis, multi-omics analysis, phospholipids, statins

## Abstract

Craniosynostosis is a common defect in the craniofacial structure of children, yet its pathogenesis remains unclear. Current research focuses mainly on bone-related genetic mutations, with less emphasis on lipid metabolism disorders. Hence, it is imperative to investigate the underlying mechanisms from various perspectives. We investigated the association between craniosynostosis and phospholipid metabolism through a multi-omics approach. This included Mendelian randomization (MR) to assess causal relationships of lipid traits, transcriptomic differential analysis of clinical samples, and single-cell RNA sequencing of murine cranial sutures to identify key cell subtypes. Furthermore, drug-target MR and network pharmacology were conducted to explore the potential therapeutic repurposing of statins. MR analysis revealed a significant protective effect of phospholipids in very low-density lipoprotein remnants against craniosynostosis. Drug-target MR targeting HMGCR indicated a protective trend (OR = 0.79) for statins, consistent with the lipid-metabolic link, although statistical significance was limited by sample size. Transcriptomic analysis identified MORN5 as a key upregulated gene enriched in glycerophospholipid metabolism. Single-cell RNA sequencing identified a specific osteogenic subtype (C4) and lipid-metabolic subtypes (C5/C6) driving the disease. Pharmacological prediction and molecular docking further suggested that Rosuvastatin could target MORN5 and epidermal growth factor receptor, showing high binding affinity (<−5 kcal/mol). Our findings provide robust evidence that phospholipid metabolism plays a critical role in craniosynostosis. While the genetic evidence for statins is exploratory, the consistent protective trend and molecular docking results suggest a potential therapeutic avenue that warrants further investigation in larger cohorts and experimental models.

## 1. Introduction

### 1.1. Skull development

Craniosynostosis is a common structural birth defect characterized by the premature fusion of 1 or more cranial sutures.^[1]^ Premature closure of these sutures impedes the normal growth of the skull. Depending on the number and type of sutures involved, it can result in cranial deformities and developmental abnormalities. In more severe cases, it can significantly impact brain development, increasing the risk of elevated intracranial pressure and disturbed cerebral blood flow. These complications may lead to developmental delays or/and learning disabilities.^[2]^ Surgical intervention is the primary treatment approach, aiming to increase cranial volume, reduce intracranial pressure, and preserve brain function. It is reported that the disease affects approximately 1 infant in every 2500 live births.^[3]^ However, the precise mechanism of craniosynostosis is not yet fully understood. Therefore, it is essential to comprehend the process of cranial suture formation and its regulatory factors for the diagnosis and treatment of craniosynostosis.

### 1.2. Phospholipids and bone development

Bone is a dynamic and active tissue that maintains mineral balance and structural integrity through constant remodeling. The reciprocal regulation between osteoblasts and osteoclasts forms the basis for bone formation and resorption during the remodeling process. Phospholipids, a type of lipids, are involved in various physiological processes in the human body and can be synthesized through multiple pathways. They are the fundamental components of the lipid bilayers in cell membranes and can be classified into several groups based on their hydrophilic head groups, including phosphatidylserine (PS), phosphatidylcholine (PC), phosphatidylethanolamine (PE), phosphatidylinositol (PI), and sphingomyelin (SM).^[4]^ Recent reports have highlighted the critical role of PS and PE on the cell surface in osteoclast fusion.^[5]^ The composition and distribution of phospholipids undergo dynamic changes, affecting the stability and fluidity of the bilayer membrane.^[6]^ Specifically, the exposure of PS on the surface of myoblast cells regulates cell fusion by preventing the formation of a nourishing layer of macrophages and syncytia in myoblast cells. It has been demonstrated that extracellular PS interacts with its receptors both in vitro between osteoclasts and in vivo between bone-resorbing cells, suggesting a potential influence on bone development.^[7]^

Craniosynostosis and osteoporosis share a genetic correlation due to their association with bone metabolism and development. Kague et al identified a gene overlap between osteoporosis and craniosynostosis by analyzing the entire human genome associated with skull bones.^[8]^ Abnormalities in lipid metabolism were detected in patients with osteoporosis using lipidomics analysis. PC, PE, and PLC, along with TG, showed alterations in osteoporosis patients, indicating a potential association between abnormal metabolism of glycerol lipids and bone remodeling and metabolism.^[9]^ These findings offer valuable guidance for future research on phospholipid metabolism and craniosynostosis.

### 1.3. Statins

Statins are commonly used lipid lowering medications, mainly prescribed to manage abnormal blood lipid levels in the body. Several studies have revealed the potential protective effects of statins on bone health, as key enzymes and molecules involved in cholesterol homeostasis are closely linked to bone formation.^[10]^ High cholesterol levels can contribute to osteoporosis, as cholesterol and its metabolites regulate the proliferation and stimulation of osteoblasts and osteoclasts, affecting bone stability. Although limited research has been conducted on statins in craniosynostosis, numerous studies on osteoporosis and fractures provide compelling evidence to suggest a potential connection between statin drugs and bone development and formation.^[11]^

In this study, we conducted a comprehensive bioinformatics analysis of craniosynostosis. This analysis included MR, single-cell sequencing, transcriptomics, and pharmacological analysis. Through these analyses, we explored the association between craniosynostosis and phospholipids. Differential analysis of different types of craniosynostosis was performed using transcriptomics techniques. Key subtypes involved in craniosynostosis development were identified through single-cell sequencing analysis. Furthermore, leveraging the lipid-metabolic link, we employed drug-target MR and network pharmacology to explore the potential repurposing value of statins as HMGCR inhibitors. While acting as an exploratory extension, this analysis aimed to uncover specific molecular mechanisms – beyond simple lipid lowering – that may intersect with the identified phospholipid pathways. These integrated analyses provide novel insights into the metabolic etiology of craniosynostosis and highlight potential therapeutic avenues for future investigation.

## 2. Materials and methods

### 2.1. Mendelian randomization

The data used in this study for MR analysis were sourced from the fifth round of data from the Finngen Biobank. This dataset consists of Genome-Wide Association Study (GWAS) data on craniosynostosis, with 405 cases and 2,18,387 controls from the European population. It includes detailed information on 4 types of craniosynostosis: Acrocephaly, Cranial suture fusion imperfection, Oxycephaly, and Trigonocephaly (detailed information can be found in the official original report). The genetic association data related to phospholipids were obtained from the IEU OpenGWAS platform, provided by Borges et al This dataset includes summary-level data from 1,15,078 European individuals, covering 4 types of lipoproteins (LDL, very low-density lipoprotein [VLDL], HDL, IDL), as well as residual lipids categorized by particle size (small, medium, large). Pharmacological Validation (Drug-Target MR): to validate the potential pharmacological effects of statins, we performed a drug-target MR analysis. Instead of using self-reported medication use, we utilized genetic variants within the HMGCR gene region (the pharmacological target of statins) to proxy HMGCR inhibition. We obtained summary statistics for LDL cholesterol from the Global Lipids Genetics Consortium (GLGC, Dataset ID: ieu-a-300). 14 independent SNPs located within ± 100 kb of the HMGCR gene (Chromosome 5) that were significantly associated with LDL levels (*P* < 5 × 10^−8^) were selected. To ensure independence, instruments were clumped at a linkage disequilibrium (LD) threshold of *r*^2^ < 0.1. The mean *F*-statistic was 146.1, ensuring robust instrument strength. Detailed information on these variants is provided in Table S1, Supplemental Digital Content, https://links.lww.com/MD/R554.

The statistical analysis was conducted using R studio (version 4.2.1; Boston). The MR R package was utilized for causal analysis between exposure and outcome, employing models such as MR Egger, Weighted median, Inverse variance weighted (IVW), and LASSO. We primarily focused on the statistical significance of the IVW results when selecting positive outcomes. Sensitivity analysis was performed using the Cochran *Q* test to assess heterogeneity among the estimated values of each single nucleotide polymorphism (SNP). A significant Cochran *Q* test indicates significant heterogeneity in the analysis results. Additionally, we used the MR-Egger intercept test to evaluate the potential horizontal pleiotropy of the SNP. A statistically significant intercept term in the MR-Egger intercept test indicates substantial horizontal pleiotropy in the MR analysis. We imposed LD criteria to mitigate the bias introduced by linkage disequilibrium (LD), requiring an *r*^2^ < 0.01 and a maximum KB of 10,000 between SNPs significantly associated with the exposure condition. Furthermore, all selected SNPs had F-statistics exceeding 10.

### 2.2. Transcriptomics analysis

RNA-seq transcriptome expression profiles and relevant clinical information (GSE27976) of craniosynostosis were obtained by searching the GEO database. Differential analysis was performed using the “R” Limma package to identify differentially expressed genes (DEGs) between different comparison groups and the control group. To ensure statistical significance, we set a false discovery rate (FDR) threshold of <0.05 and a fold change threshold of 1.5-fold. This study aims to evaluate the impact of differential gene analysis on enrichment pathways and their underlying molecular mechanisms.

### 2.3. Single-cell analysis

The single-cell RNA sequencing data of GSE138881 was obtained from the Gene Expression Omnibus (GEO) gene expression database. The R package “Seurat” is utilized for canonical correlation analysis (CCA) to mitigate batch effects. We isolated and analyzed the skeletal stem/progenitor cells from the parietal bone (PF), sagittal suture (SAG), and coronal suture (COR) of 3-day-old mice that were freshly harvested. Our analysis focused on the tibial growth plate (GP), involving 2 cases of COR, 2 cases of GP, 2 cases of SAG, 2 cases of PF, and 1 case of tibial tissue.^[12]^ Cell classification was performed using a module optimization method. For visualization purposes, we employed the uniform manifold approximation and projection algorithm to map cells onto a 2-dimensional plane, enabling us to explore the biological associations between cells. Each cell is represented as a point in the clustering plot, with cells closer in space indicating more similar gene expression patterns. Based on this principle, cells were classified into distinct subtypes.

A collection of mouse Gene Ontology (GO) pathways related to phospholipid metabolism and bone formation (version 7.3) from the Molecular Signatures Database (MSigDB, http://www.gsea-msigdb.org/) was downloaded to investigate the functional differences among different subtypes of tumor cells. Functional enrichment analysis was then performed on each cell subtype using the gene set variation analysis (GSVA) algorithm.

The R package “Monocle3” (version 1.0.1) was employed to explore the dynamics of cell subtypes and their clinical relevance. Subsequently, the R package CellChat (version 1.1.3) was utilized to analyze the intercellular communication network and investigate the interactions among 6 cell types.

### 2.4. Pharmacological analysis

The Simplified Molecular Input Line Entry System (SMILES) code of Rosuvastatin was obtained from the PubChem database (https://pubchem.ncbi.nlm.nih.gov). This code was then used to predict potential targets through structural similarity analysis in the SwissTargetPrediction database (http://www.swisstargetprediction.ch). The search was performed in the GeneCards (https://www.genecards.org/) and OMIM (https://www.omim.org/) databases to identify disease-associated targets. After removing duplicates and merging the results, the common targets between Rosuvastatin active compounds and diseases were considered potential key targets for therapeutic intervention. The Cytoscape 3.9.0 software was employed to analyze the network topology by importing the relevant files. Targets were adjusted in terms of graphical representation, color, transparency, and size based on the degree value, which represented the number of gene connections, allowing the construction of a “drug-ingredient-target” network graph. Furthermore, the intersecting genes were input into the String online analysis software (https://string-db.org/) to explore protein–protein interactions (PPIs). The top 10 core targets were selected based on their Degree values. Finally, the GO and Kyoto Encyclopedia of Genes and Genome (KEGG) functional enrichment analyses elucidated the main signaling pathways involved in the therapeutic effects of the drug on diseases. The 3D crystal structures of the target proteins (MORN5, epidermal growth factor receptor [EGFR], MAPK1, ACTB) were retrieved from the RCSB Protein Data Bank (PDB). The chemical structure of Rosuvastatin was obtained from the PubChem database. The receptor proteins were prepared by removing water molecules and adding polar hydrogens using AutoDock Tools. Molecular docking simulations were performed using AutoDock Vina software to calculate the binding affinities. The grid box was centered on the active site of each receptor. Finally, the docking results were visualized and analyzed using BIOVIA Discovery Studio Visualizer, which generated both the 3D binding poses and the 2D interaction diagrams to identify key amino acid residues and specific interaction types (e.g., hydrogen bonds).

## 3. Results

### 3.1. Association between lipoproteins, VLDL remnants, and rosuvastatin with craniosynostosis risk

**Figure 1.**
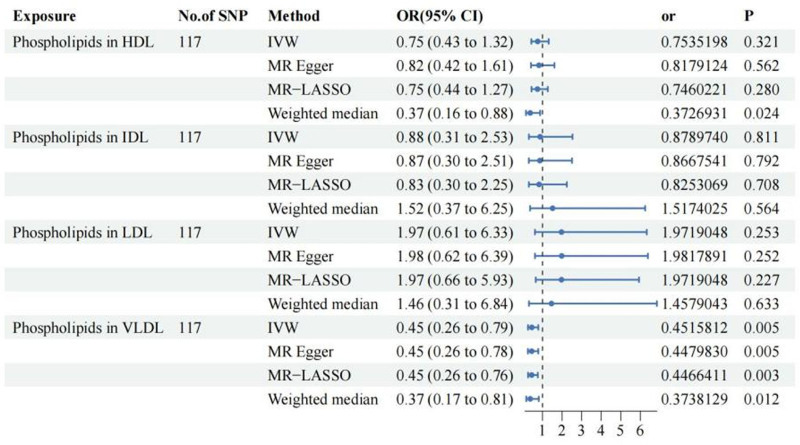
Forest plot summarizing the Mendelian randomization estimates for the causal effect of phospholipids in lipoprotein particles on craniosynostosis risk. The forest plot displays the odds ratios (OR) and 95% confidence intervals (CI) for phospholipids in high-density lipoprotein (HDL), intermediate-density lipoprotein (IDL), low-density lipoprotein (LDL), and very low-density lipoprotein (VLDL) particles. Instrumental variables: the analysis utilized 117 SNPs as instrumental variables for each exposure. Methods: results are shown for the primary inverse variance weighted (IVW) method, along with sensitivity analyses using MR Egger, MR-LASSO, and weighted median methods. Visual key: the *x*-axis represents the OR on a linear scale. Blue horizontal lines represent the 95% CI, and the central markers indicate the OR point estimates. The vertical dashed line at OR = 1 indicates the null value; intervals crossing this line are not statistically significant. *P*-values < .05 indicate statistical significance (e.g., consistent protective associations observed for VLDL phospholipids). CI = confidence interval, HDL = high-density lipoprotein, IDL = intermediate-density lipoprotein, IVW = inverse variance weighted, LDL = low-density lipoprotein, MR = Mendelian randomization, MR-LASSO = Mendelian randomization-least absolute shrinkage and selection operator, OR = odds ratio, SNP = single nucleotide polymorphism, VLDL = very low-density lipoprotein.

**Figure 2.**
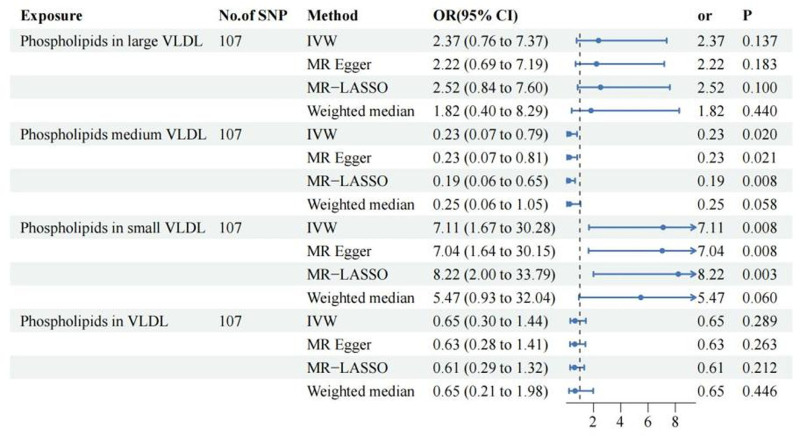
Forest plot of Mendelian randomization estimates for the causal effect of phospholipids in VLDL particles of different sizes on craniosynostosis risk. The forest plot displays the causal effect estimates for Phospholipids in large VLDL, medium VLDL, small VLDL, and total VLDL particles. Instrumental variables: a total of 107 SNPs were utilized as instrumental variables for each exposure. Methods: results are presented for the primary inverse variance weighted (IVW) method, alongside sensitivity analyses using MR Egger, MR-LASSO, and weighted median methods. Visual Key: the *x*-axis represents the odds ratio (OR) on a linear scale. Blue horizontal lines represent the 95% confidence intervals (CI), and the central markers indicate the point estimates for the OR. The vertical dashed line at OR = 1 indicates the null value. Interpretation: Significant associations were observed for specific particle sizes: medium VLDL phospholipids showed a protective effect (OR < 1, *P* < .05), while small VLDL phospholipids were associated with an increased risk (OR > 1, *P* < .05). No significant causal associations were found for large VLDL or total VLDL phospholipids. CI = confidence interval, IVW = inverse variance weighted, MR = Mendelian randomization, MR-LASSO = Mendelian randomization-least absolute shrinkage and selection operator, OR = odds ratio, SNP = single nucleotide polymorphism, VLDL = very low-density lipoprotein.

Initially, the relationship between phospholipids in 4 lipoproteins and the occurrence of craniosynostosis was explored, revealing no strong correlation between phospholipids in HDL, IDL, and LDL lipoproteins and the risk of craniosynostosis. However, there is a negative association between phospholipids in VLDL lipoproteins and the risk of craniosynostosis (OR: 0.45, 95% CI: 0.26–0.79, *P* = .005). This association remains consistent across the LASSO, MR Egger, and Weighted median analyses (Fig. 1). Additionally, we further investigated the relationship between the phospholipid content in VLDL remnants within VLDL and craniosynostosis. We found that phospholipids in Medium VLDL act as a protective factor against craniosynostosis (OR: 0.23, 95% CI: 0.07–0.79, *P* = .02), and this association is supported by the results of the other 3 methods (Fig. 2). Using dual-sample MR analysis, To verify the causal relationship, we conducted a drug-target MR analysis using 14 validated SNPs in the HMGCR region. The analysis revealed a protective direction for HMGCR inhibition (IVW OR = 0.79; 95% CI: 0.39–1.62). While this association did not reach statistical significance (*P* = .53) likely due to the limited sample size of the craniosynostosis cohort (N = 405), the effect estimate suggests a potential risk reduction, which aligns with the protective role of lipid modulation observed in our VLDL analysis. Given this consistent biological trend, we proceeded to investigate the underlying molecular mechanisms using network pharmacology.

### 3.2. Elevated expression of MORN5 and glycerophospholipid metabolism pathway enrichment in craniosynostosis

**Figure 3.**
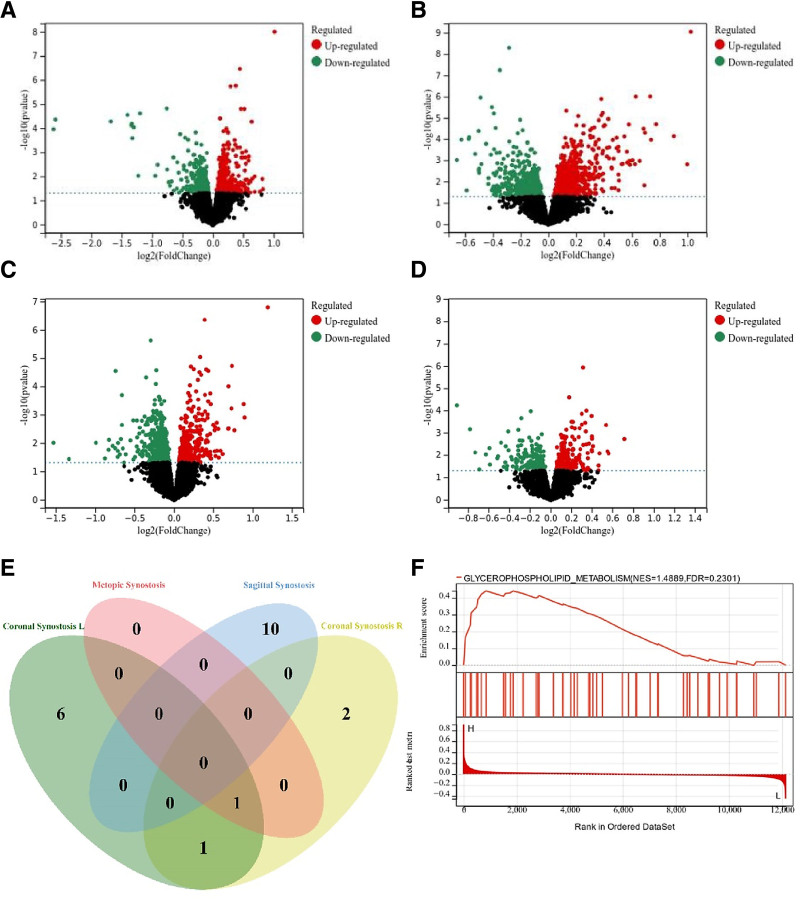
Volcano plot showing differences in (A) left coronal suture premature closure compared to the normal group; (B) right coronal suture premature closure compared to the normal group; (C) frontal suture premature closure compared to the normal group; (D) sagittal suture premature closure compared to the normal group. (E) Venn diagram depicting differential genes among 4 distinct types of cranial suture premature closure. (F) Enrichment analysis of single-gene GSEA pathways. GSEA = gene set enrichment analysis.

A dataset (GSE27976) about craniosynostosis was obtained in this study. We compared transcriptome RNA expression data from 199 patients, including 100 with sagittal craniosynostosis, 50 with coronal craniosynostosis, 49 with metopic craniosynostosis, and 50 in the control group.^[13]^ We performed a differential analysis comparing the early closure of different types of cranial sutures with normal samples (Fig. 3). Our differential analysis identified numerous DEGs across the subtypes, as visualized in the volcano plots (Fig. 3A–D). To ensure target specificity, we employed a strict prioritization strategy. First, we filtered for genes that were consistently upregulated across all 3 distinct subtypes (right coronal, left coronal, and metopic synostosis), distinguishing common pathogenic drivers from subtype-specific variations. Among these consistent DEGs, MORN5 was prioritized because it satisfied 3 key criteria – Consistency: it was universally upregulated across all subtypes; Mechanistic relevance: single-gene analysis using gene set enrichment analysis positioned it specifically within the glycerophospholipid metabolism pathway (Fig. 3F); and Druggability: it demonstrated high binding potential with statins in our pharmacological analysis. This convergence suggests MORN5 is a functional driver rather than a random transcriptomic artifact.

### 3.3. Analysis of subtype characteristics and interactions in cranial suture formation

The cranial sutures in mice and humans exhibit similarities. Both of them have paired anterior frontonasal sutures (AF) located between the frontal bones, paired posterior frontonasal sutures (PF) resembling the human metopic suture, coronal sutures (COR) situated between the frontal and parietal bones, paired sagittal sutures (SAG) between the parietal bones, and the lambdoid suture (LAM) between the occipital and parietal bones. The GEO database was utilized to obtain single-cell RNA profiles of rat skeletal stem cells isolated from the parietal bone (PF), sagittal suture (SAG), and coronal suture (COR).

#### 3.3.1. Identification of key cell subtypes

**Figure 4.**
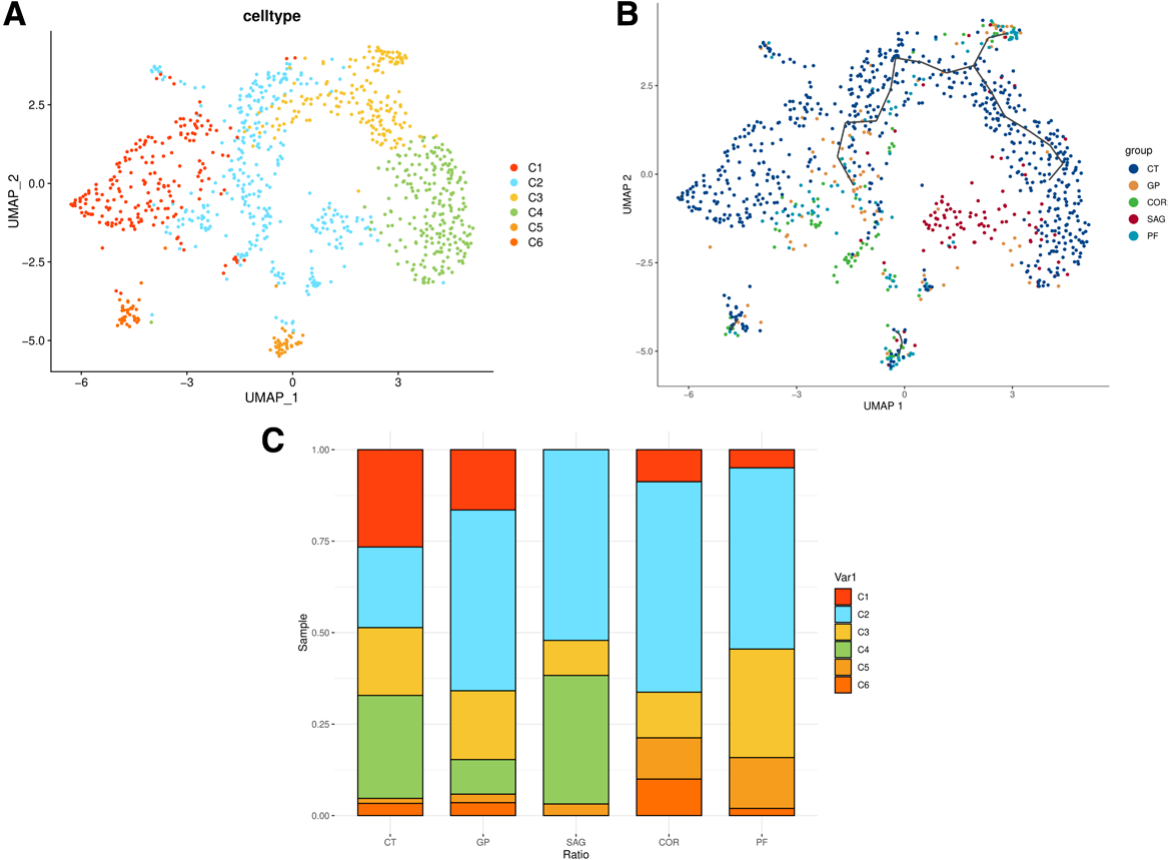
Single-cell transcriptomic landscape and heterogeneity of cranial suture progenitors. (A) Uniform manifold approximation and projection (UMAP) visualization of 1018 single cells that passed quality control (nFeature_RNA: 300–5000; percent_mito < 5%). Cells are colored by cell subtype (C1–C6), identified via graph-based clustering (Seurat, Resolution = 0.8, PCs = 15). (B) UMAP projection colored by tissue of origin, distinguishing cells from the coronal suture (COR), sagittal suture (SAG), parietal bone (PF), growth plate (GP), and tibia (CT). (C) Stacked bar charts showing the cellular composition of each tissue. Note the specific enrichment of the osteogenic C4 subtype in limb bones (GP, CT) compared to the prevalence of lipid-metabolic subtypes in cranial sutures. Cluster Annotation: clusters were annotated based on the expression of canonical marker genes: C1 is defined by S100a6 and Tppp3; C3 is characterized by the Wnt-inhibitor Wif1; C4 is marked by osteochondrogenic genes (Col2a1, Hapln1); while C5 and C6 are distinguished by lipid-metabolic markers (e.g., Smpd3, Agpat6). UMAP = uniform manifold approximation and projection.

Dimensionality reduction and clustering on all cells were performed using the uniform manifold approximation and projection analysis method, resulting in the categorization of cells into 6 subtypes (C1–C6; Fig. 4). Our cell proportion analysis revealed the expression of the C4 subtype in the tibia (CT) and bone marrow derived from GP and SAG. However, no expression was observed in bone marrow derived from the other 2 sources. No expression of the C1 subfamily was detected in the osteoblast stem cells derived from SAG.

**Figure 5.**
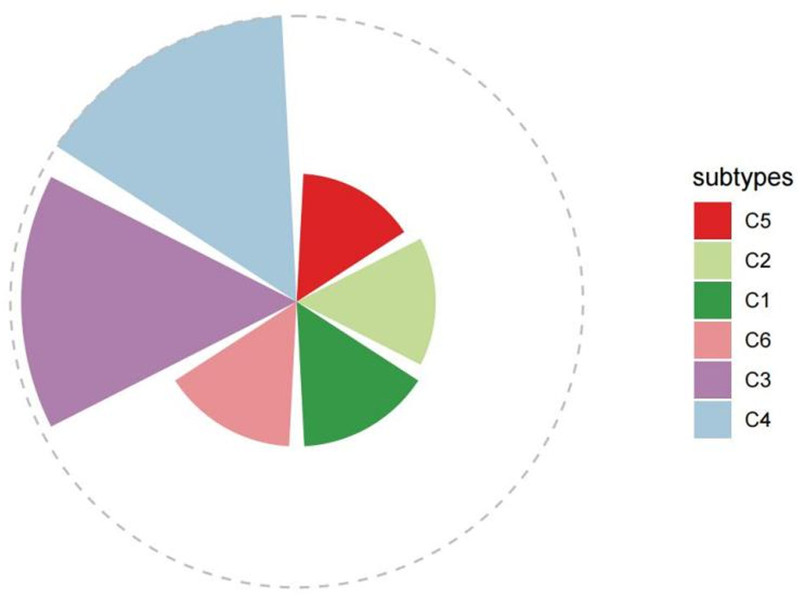
Evaluation of the functional contribution of cell subtypes to craniosynostosis. A circular bar plot quantifying the contribution scores of each cell subtype (C1–C6) to the disease phenotype. Calculation of contribution scores: the contribution score was calculated based on the top 100 upregulated differentially expressed genes (DEGs) identified between the disease and control groups. For each cell subtype, the score represents the mean value of the geometric mean of the fold change in expression (FC_exp_) and the fold change in the proportion of expressing cells (FC_prop_) for these signature genes. Conclusion: the C4 subtype exhibits the highest contribution score, identifying it as the primary cell population potentially driving the pathogenic process of cranial suture closure. DEG = differentially expressed gene.

**Figure 6.**
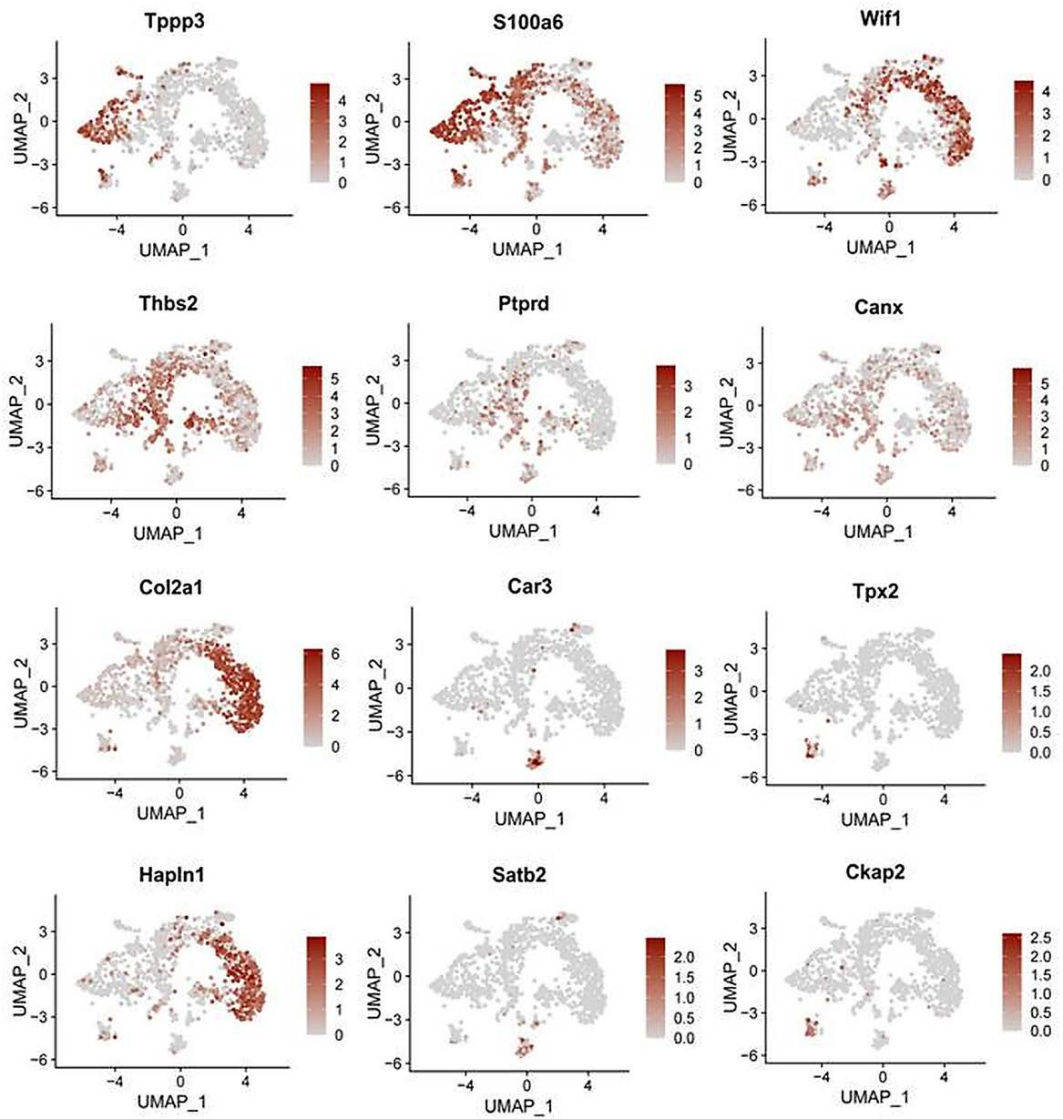
UMAP projection of marker genes for each subtype, with gene expression levels indicated by color. UMAP = uniform manifold approximation and projection.

The impact of each subtype on cranial suture formation was evaluated, indicating a more significant contribution in the C4 subtype than in other subtypes (Fig. 5). Significant marker genes for each subtype were successfully identified. Specifically, WIF1 was identified as the signature gene for the C3 subtype, while Col2a1 and Hapln1 were identified as signature genes for the C4 subtype. The C1 cell subtype exhibited significant expression of S100a6 and TPPP3 genes (Fig. 6).

**Figure 7.**
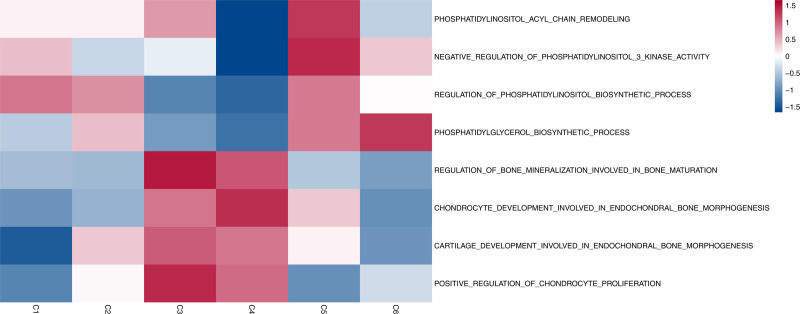
Landscape of metabolic and osteogenic pathway enrichment across cranial suture stem cell subtypes. The heatmap visualizes the normalized enrichment scores (NES) calculated via gene set variation analysis (GSVA) using gene sets retrieved from the MSigDB database (v7.3). The color gradient represents the magnitude of pathway regulation, ranging from blue (negative enrichment/downregulation) to red (positive enrichment/upregulation). Statistical significance of pathway differences between subtypes (e.g., C3/C4 vs C5/C6) was determined using Limma-moderated *t* tests (adjusted *P* < .05). Notably, the C3/C4 subtypes exhibit a distinct osteogenic profile characterized by high enrichment of regulation of bone mineralization and chondrocyte development pathways. Conversely, the C5/C6 subtypes display a specific lipid-metabolic profile, marked by the significant activation of phosphatidylinositol biosynthetic processes, sphingolipid metabolism, and glycerophospholipid regulation, supporting a metabolic-regulatory role in suture patency.

Subsequently, GSVA was performed to conduct an enrichment analysis of pathways related to bone formation and phospholipid metabolism in each subtype (Fig. 7). The analysis revealed a complex metabolic landscape where osteogenic differentiation is intrinsically coupled with specific lipid remodeling. Detailed stratification demonstrated that while both C3 and C4 were enriched in canonical bone mineralization pathways (normalized enrichment score [NES] > 1.8, *P* < .01), the C4 subtype exhibited a unique dual-identity. Validated by trajectory analysis, C4 was defined by high-ranking chondrogenic drivers such as Col2a1 (Moran’s *I* = 0.70) and Hapln1 (Moran’s *I* = 0.58). Crucially, unlike the canonical osteoblasts (C3), the C4 subtype showed specific enrichment for “Phosphatidylinositol acyl-chain remodeling” (NES > 1.5, *P* < .05). This integration of matrix synthesis and phospholipid remodeling suggests that C4 represents a chondrogenic-like osteoblast population engaging in lipid-dependent endochondral ossification (e.g., matrix vesicle formation).

In distinct contrast, the C5 and C6 subtypes were defined by a robust upregulation of glycerophospholipid and sphingolipid metabolic networks. Specifically, pathways such as “Phosphatidylinositol acyl chain remodeling” and “Sphingolipid metabolic process” showed significant positive enrichment (NES > 1.5, *P* < .05). To validate the biological specificity of this signature, we examined trajectory-dependent gene expression and identified Smpd3 (Sphingomyelin Phosphodiesterase 3, Moran’s *I* = 0.37) and Agpat6 (1-Acylglycerol-3-Phosphate O-Acyltransferase 6, Moran’s *I* = 0.24) as critical metabolic regulators distinguishing the C5/C6 lineage. These findings provide rigorous molecular evidence that the C5/C6 subtypes function as a specialized lipid-metabolic niche within the cranial suture microenvironment.

#### 3.3.2. Identification of the transcriptomic characteristics of the C4 subtype

**Figure 8.**
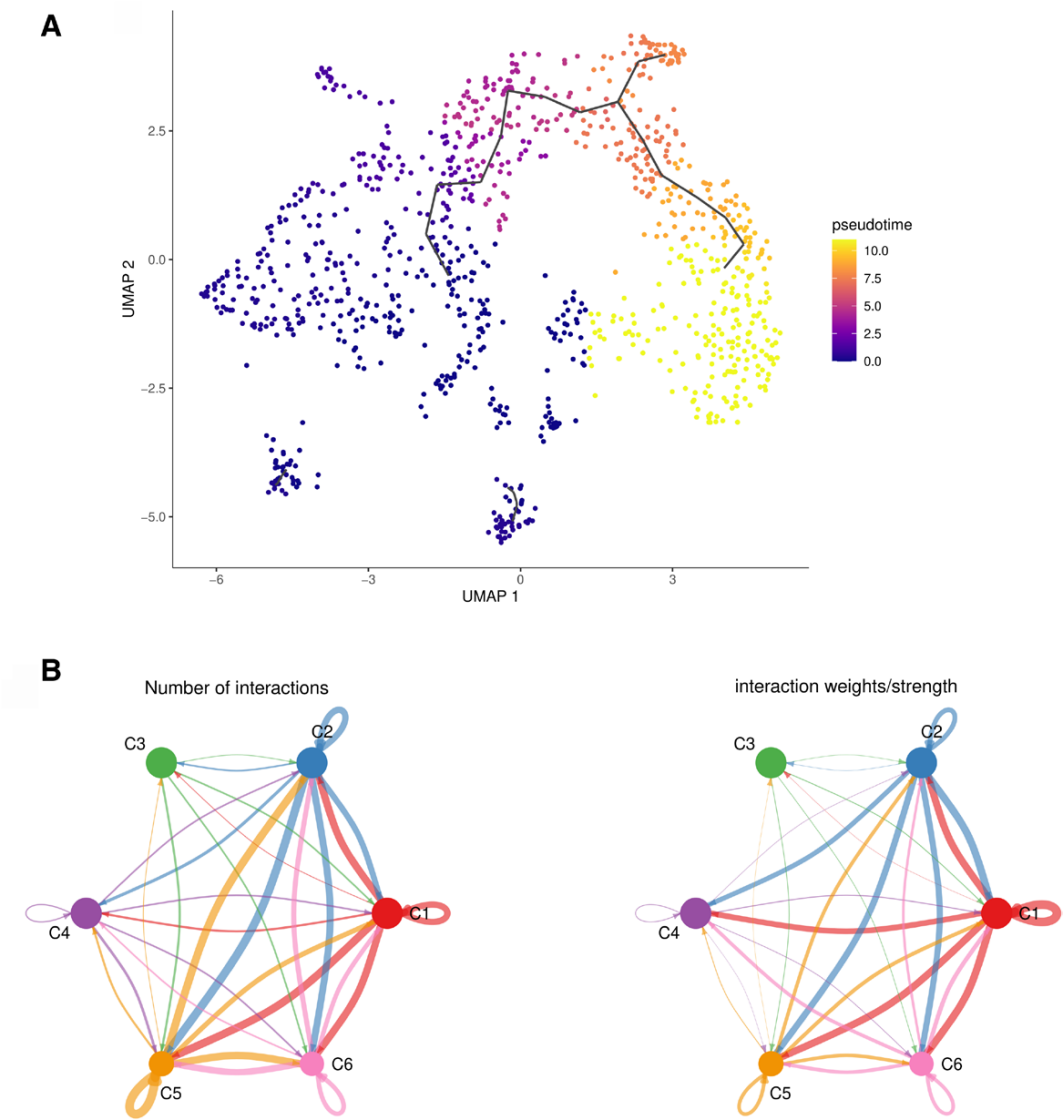
(A) UMAP projection of epithelial cells with pseudotime-based color, where purple represents earlier pseudotime and orange represents later pseudotime. (B) Interaction count (left) and strength (right) of the cell communication network among osteoblast subtypes. UMAP = uniform manifold approximation and projection.

Pseudotime analysis and cell communication analysis was employed to explore the transcriptomic characteristics of the C4 subtypes, which are associated with osteogenesis. The results revealed that the C4 subfamily is in the latest pseudotime stage and originates from the differentiation of the C3 subtype. We generated a marker expression atlas for C4 and observed a highly consistent trend in the temporal fluctuations of core marker genes with the pseudotime trajectory. Moreover, cellular communication analysis was conducted to investigate the interactions among osteogenic stem cells, exhibiting a reduced cellular communication frequency in the C3 and C4 subtypes (Fig. 8).

### 3.4. Identification of potential targets and pathways for Rosuvastatin in the treatment of craniosynostosis

**Figure 9.**
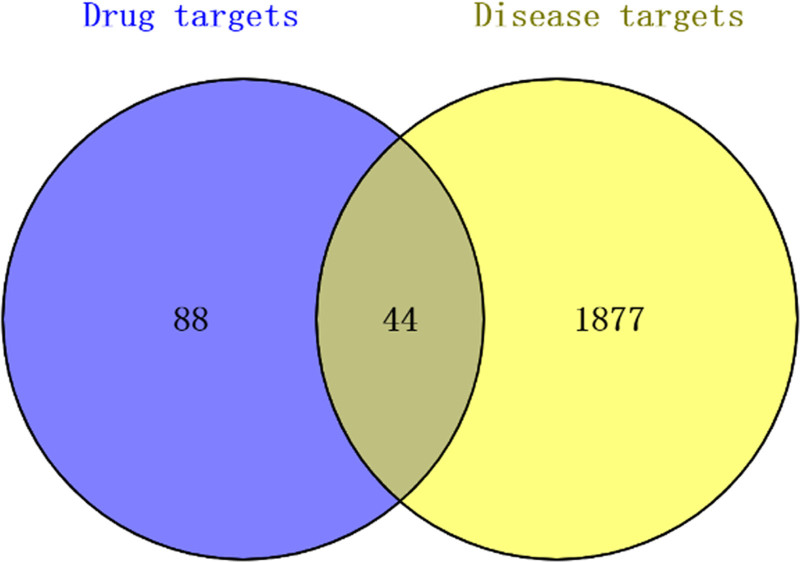
Venn diagram of the intersection targets between Rosuvastatin and craniosynostosis.

A total of 88 potential targets for the active ingredients were identified through a comprehensive search of the PubChem database. A total of 1877 target genes related to craniosynostosis were identified based on GenCards and the OMIM database. We found 44 common targets in both datasets by mapping with the 88 potentially active components (Fig. 9). These targets may be potential candidates for the treatment of craniosynostosis using Rosuvastatin.

**Figure 10.**
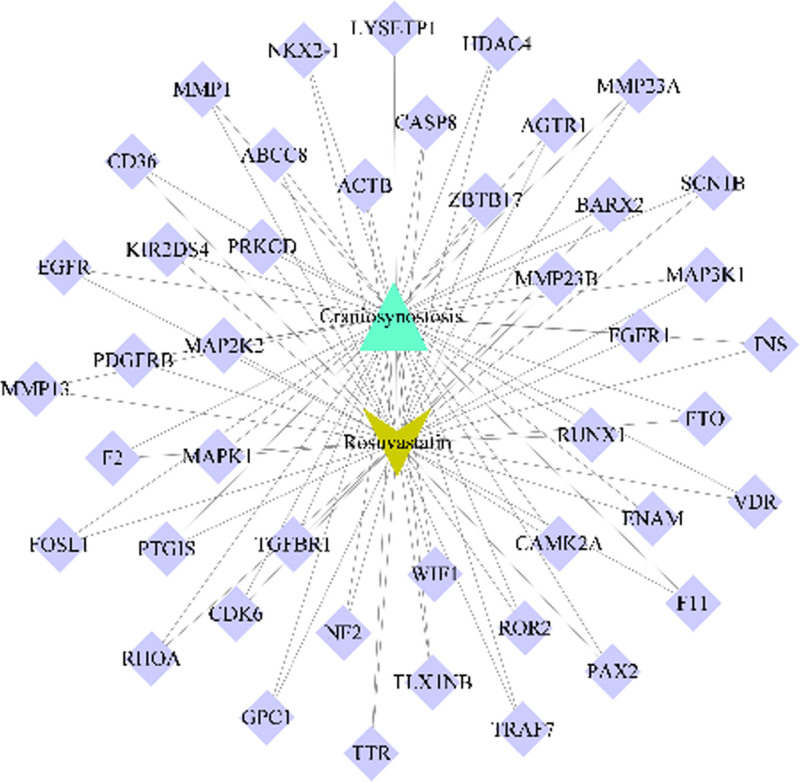
Network visualization of the “Drug-Ingredient-Target” interactome. The yellow V-shape represents Rosuvastatin; the cyan triangle represents Craniosynostosis; purple diamonds represent overlapping gene targets. Edges indicate interaction relationships.

**Figure 11.**
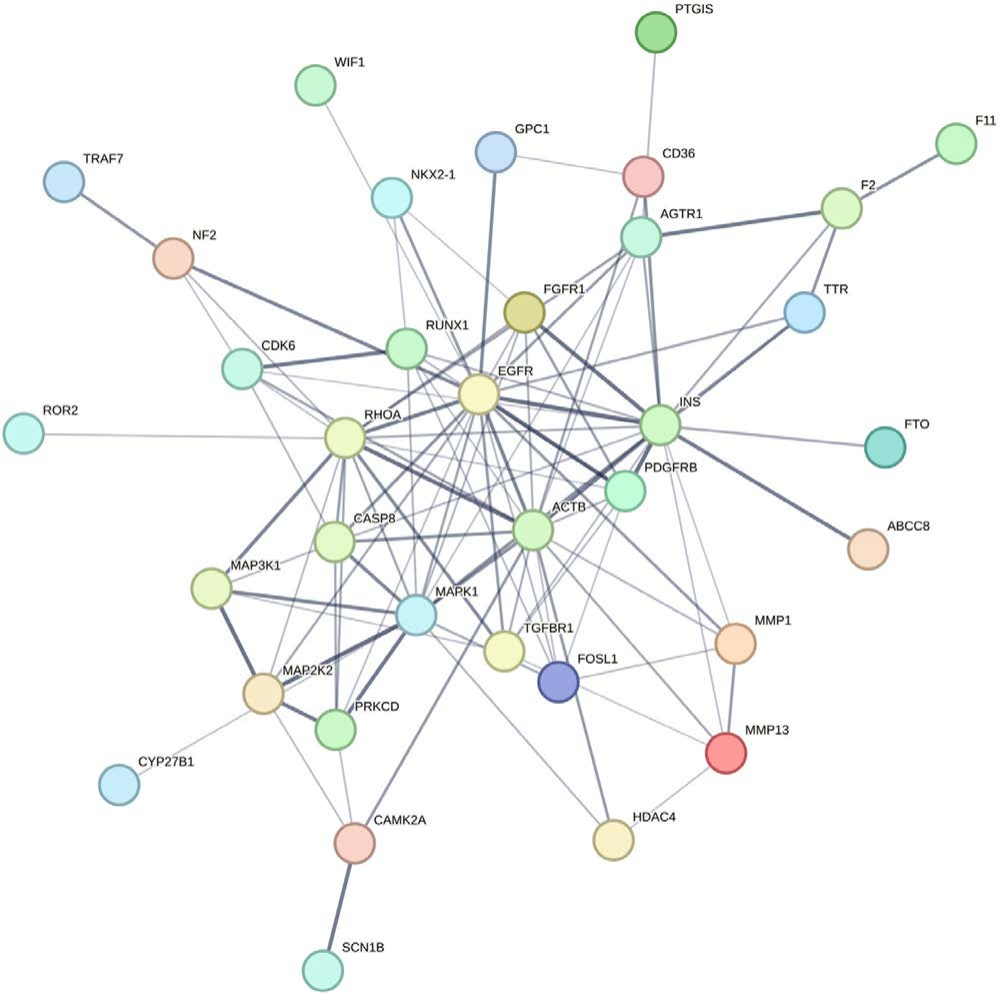
Protein–Protein Interaction (PPI) network of overlapping targets. Nodes represent proteins, and edges represent functional interactions. Node size and color gradient correspond to the Degree value, with larger/darker nodes (e.g., EGFR, MAPK1) indicating a higher number of connections (Hub genes). PPI = Protein–Protein Interaction.

**Figure 12.**
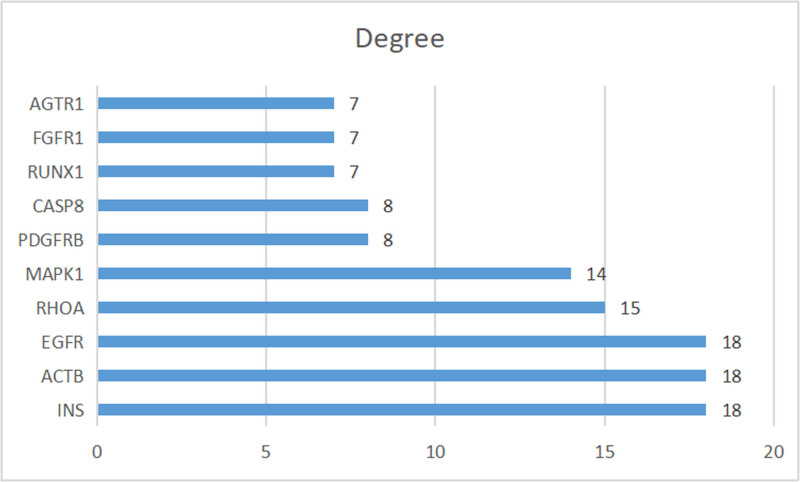
Key target protein selection diagram for rosuvastatin treatment of craniosynostosis.

A network graph was constructed to illustrate the relationships between the screened active compounds, targets, and genes associated with craniosynostosis (Fig. 10). Additionally, a PPI network graph of the overlapping targets was created using the String online platform (Fig. 11). The drug targets and network targets were mapped and filtered in Cytoscape 3.9.0 based on their degree, betweenness, and closeness centrality values to conduct 3 rounds of topological analysis and construct a core target protein graph. The top 10 hub genes were selected based on their degree values (Fig. 12). Among these core targets, EGFR (Degree = 18) was identified as a central hub. EGFR is biologically relevant to our hypothesis as it is known to localize within lipid rafts (membrane microdomains enriched in cholesterol and sphingolipids). Its signaling activity, which regulates osteoblast differentiation and cranial suture closure, is strictly modulated by the membrane lipid composition. This topological centrality suggests that Rosuvastatin may exert its effects by modifying the lipid environment surrounding EGFR, thereby attenuating pathological signaling.

**Figure 13.**
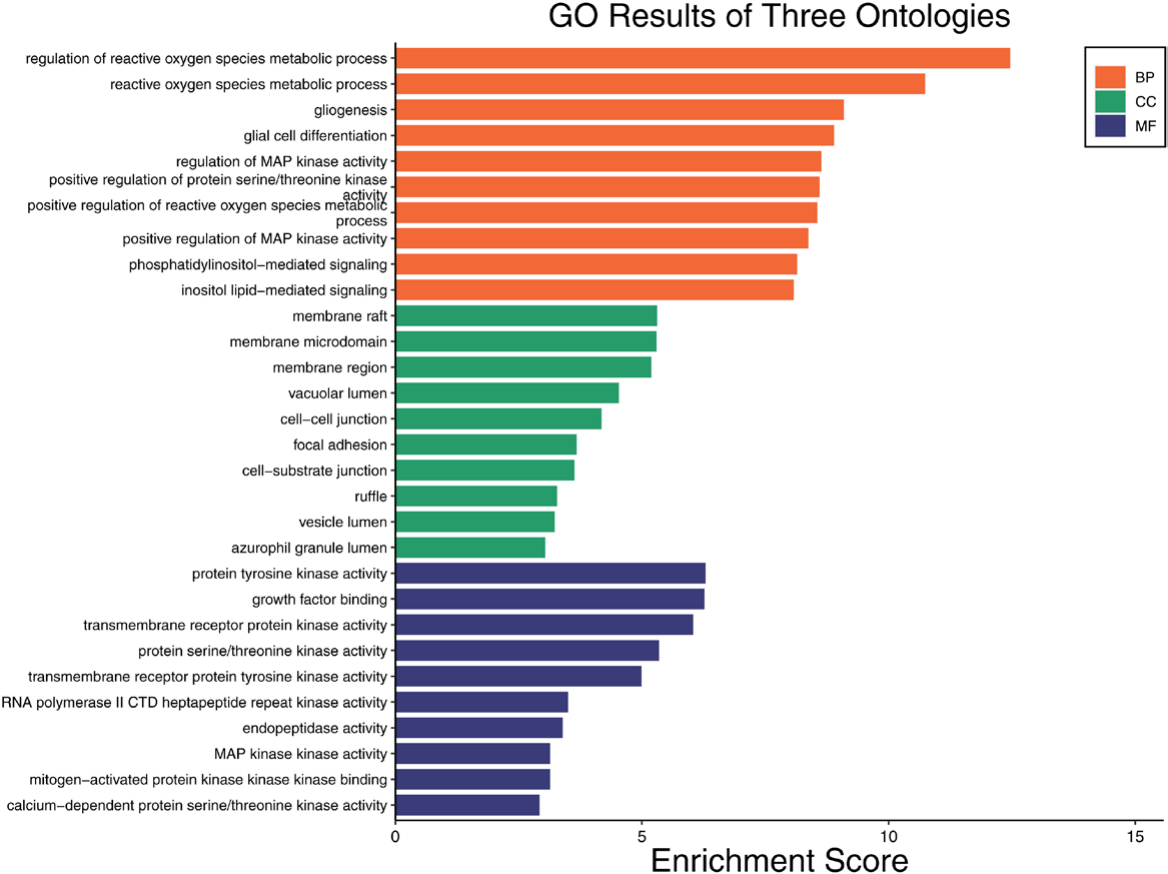
GO enrichment analysis of targets for craniosynostosis treatment by active ingredients of Rosuvastatin. GO = Gene Ontology.

**Figure 14.**
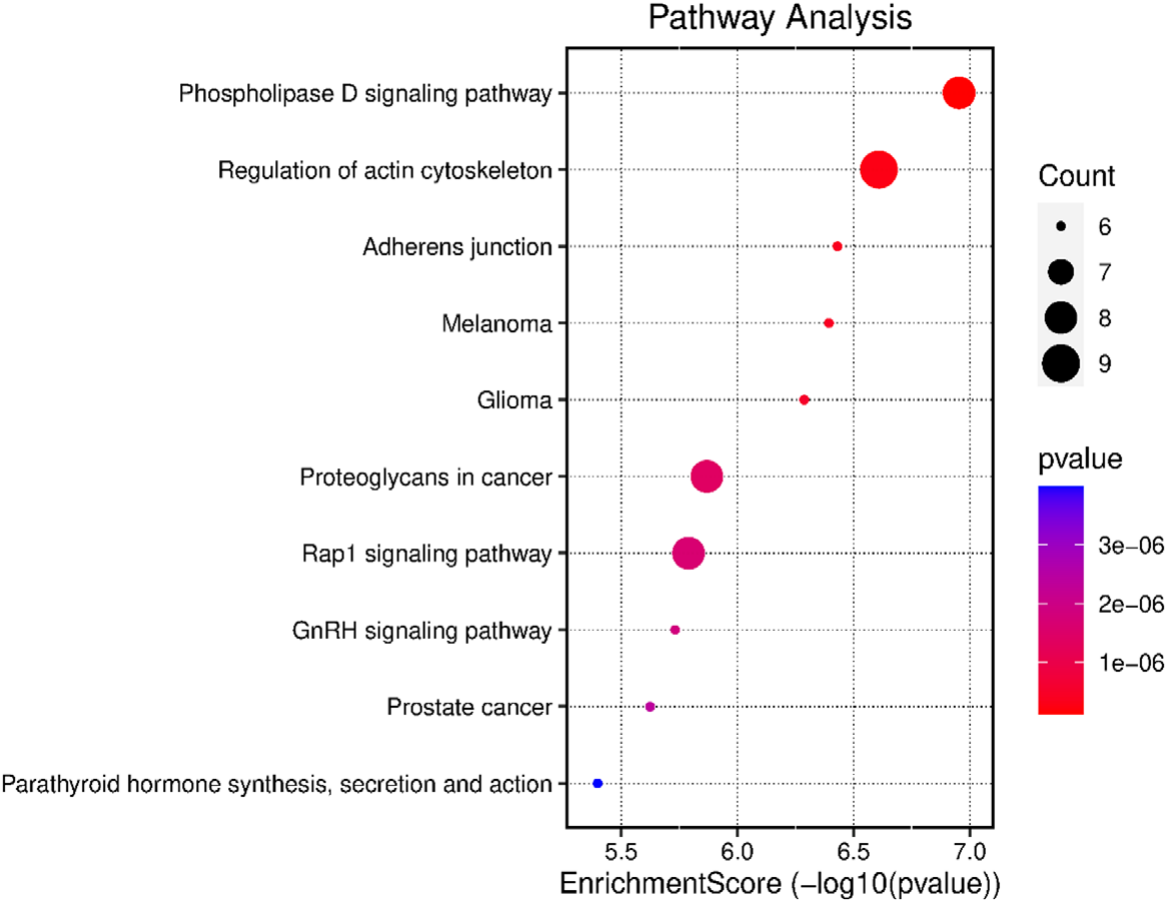
Enrichment bubble map of KEGG pathways. KEGG = Kyoto Encyclopedia of Genes and Genomes.

Enrichment analysis of potential target genes was conducted using GO and the KEGG. The results indicated that the GO enrichment analysis primarily involved biological processes such as apoptosis, phosphatidylinositol metabolism, glial cell regeneration, and inositol lipid metabolism (Fig. 13). The cellular components mainly implicated included membrane rafts, neuronal cell bodies, and plasma membranes. The molecular functions identified were primarily related to protein serine/threonine/tyrosine kinase activity. The KEGG pathway enrichment analysis revealed 44 intersecting target genes involved in various pathways. Notably, the phospholipase D and actin cytoskeleton signaling pathways were identified as the primary ones (Fig. 14).

**Figure 15.**
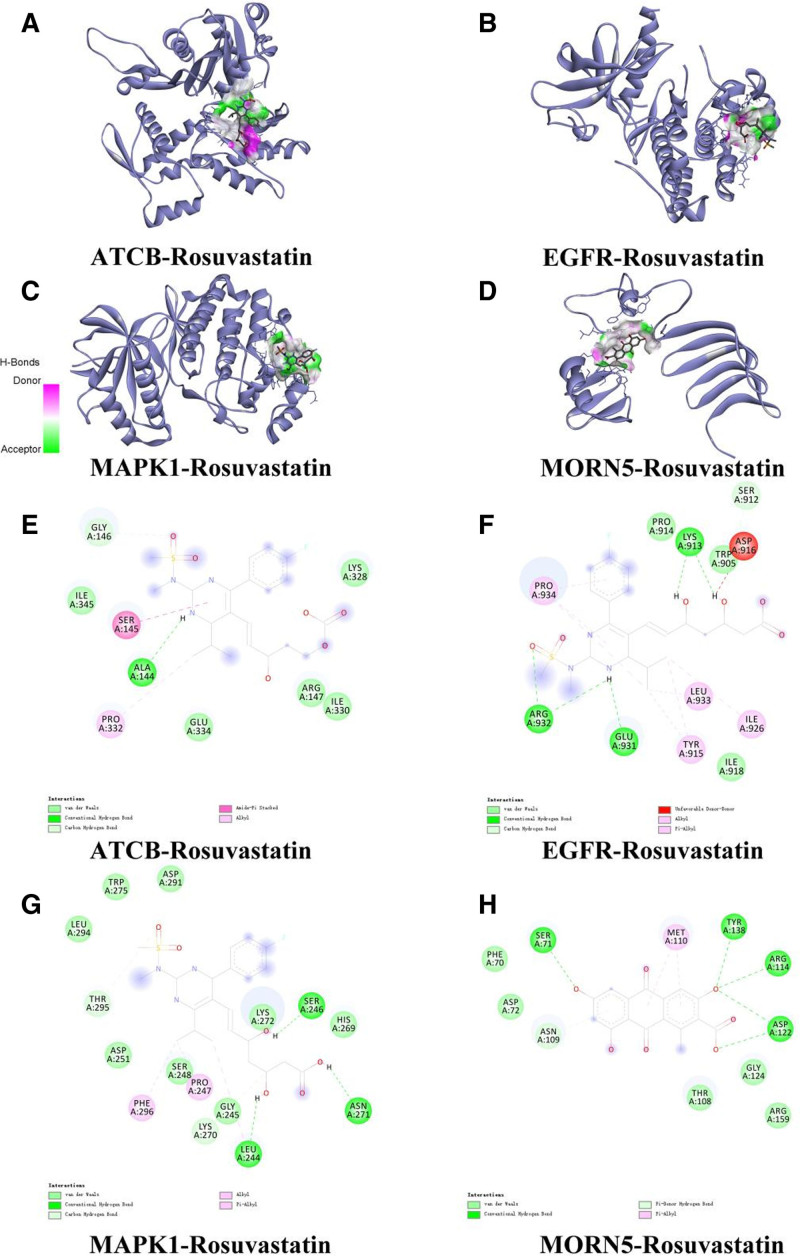
Visualization of molecular docking between Rosuvastatin and core targets. (A–D) 3D surface representation showing the binding pocket of ACTB, EGFR, MAPK1, and MORN5 with Rosuvastatin. (E–H) 2D interaction diagrams detailing the specific amino acid residues involved in binding. Green circles indicate Van der Waals interactions; pink/purple circles indicate Pi-alkyl or Pi-sigma bonds; light green circles indicate Hydrogen bonds. All docking simulations were performed using AutoDock Vina.

It is widely recognized that the stronger the affinity between a ligand and a receptor, the lower the energy required, thus increasing the likelihood of interaction. Molecular Docking Analysis The binding affinities between Rosuvastatin and its targets were calculated and are listed in Table 1. Rosuvastatin exhibited strong binding potential with MORN5 (-6.56 kcal/mol) and moderate affinity with EGFR (-4.77 kcal/mol). Notably, the binding energy for MORN5 surpassed the standard threshold of −5.0 kcal/mol, indicating a highly stable interaction. Detailed 2D interaction diagrams (Fig. 15E–H further revealed the structural basis of these interactions: Rosuvastatin forms stable hydrogen bonds with specific residues, such as Arg 123, Lys 125, and Glu 150 in MORN5, and Arg 331, Glu 331, and Pro 334 in EGFR. Comparison with the reference protein ACTB (binding energy listed in Table 1) further validated the specificity of these interactions. These specific molecular interactions suggest that Rosuvastatin may exert its biological effects by directly modulating the activity of these key proteins.

**Table 1:** Molecular docking binding energies.

Compound	Binding energy/(kcal/mol)			
	ACTB	EGFR	MAPK1	MORN5
Rosuvastatin	−4.68	−4.77	−5.87	−6.56

## 4. Discussions

The upregulation of MORN5 observed in our study is likely a functional driver of pathological ossification rather than a secondary effect. Cela et al previously established that MORN5 is indispensable for craniofacial development, functioning as a modulator of the BMP and TGF-β signaling cascades. Specifically, they demonstrated that MORN5 knockdown leads to the derepression of critical osteogenic factors, including GDF2 (BMP9), RUNX1, and TGFBR2.^[14]^ Crucially, the authors noted that MORN motifs are characteristic of the phosphatidylinositol phosphate kinase family,^[14]^ suggesting a lipid-dependent mechanism of action. Integrating this with our single-gene analysis using gene set enrichment analysis results – which showed significant enrichment of MORN5 in glycerophospholipid metabolism – we propose a novel mechanistic hypothesis: MORN5 may function as a membrane-associated scaffold that alters the local phosphatidylinositol composition. Since canonical BMP/TGF-β receptors rely on specific lipid microenvironments (e.g., lipid rafts) for stability and signal transduction, we postulate that MORN5 upregulation creates a hyper-responsive osteogenic membrane environment. This would amplify BMP/TGF-β signaling loops (involving BMP9 and RUNX1), thereby accelerating osteoblast differentiation and premature suture fusion. The parietal foramen (PF), one of the early healing cranial sutures, inhibits intramembranous ossification by continuously activating the Wnt signal, ultimately leading to suture closure.^[15]^ It can be found that the expression of the C4 subtype was absent in the skeletal stem cells derived from PF. Notably, the genes hyaluronic acid and proteoglycan link protein 1 (HAPLN1) were significantly expressed in the C4 subtype, indicating their involvement in osteoblast differentiation and regulation of genes associated with cartilage regeneration. This finding suggests that the C4 subtype may be crucial in promoting bone development. In contrast, the Wnt signaling pathway potential inhibitor WIF1 exhibited increased expression in the C3 subtype, suggesting its role in inhibiting this pathway.^[16]^ No significant expression differences were observed in the C4 subtype during further chondrogenesis. Li et al demonstrated that the downregulation of S100a6 enhances the osteogenic differentiation activity of mesenchymal stem cells.^[17]^ Additionally, S100A6 knockdown enhances BMP9-induced osteogenesis, while S100A6 overexpression inhibits osteogenic differentiation. The significantly increased expression of S100a6 in the C1 subtype, along with the delayed closing time of the SAG gene, indicates a loss of expression in the C1 subtype. Therefore, the C1 subtype may be crucial in promoting osteogenic differentiation and delaying the closure of cranial sutures. Mechanistic Interpretation of Cellular Communication Interestingly, our CellChat analysis (Fig. 8) showed that the C4 subtype exhibits reduced intercellular communication compared to stem-like progenitors (C1/C2). We postulate that this reflects a functional state transition. As cells commit to the chondrogenic-like lineage (C4), they likely shift resources from paracrine signaling (broadcasting) to intracellular metabolic reprogramming. The synthesis of collagen matrix (indicated by Col2a1) and the remodeling of membrane lipids (indicated by Phosphatidylinositol pathways) are energy-intensive processes required for matrix vesicle formation. Therefore, the ‘low communication’ profile of C4 signifies a specialized state where cellular machinery is dedicated to matrix mineralization and membrane remodelingrather than intercellular signaling.

Lipid signaling and membrane lipid composition are critical for skeletal development and maintenance. Previous studies have demonstrated the involvement of lipids in regulating osteoclast/osteoblast functions mediated by Wnt signaling.^[18,19]^ Imbalances in lipid metabolism have been associated with various skeletal and joint disorders, with different lipid profiles observed in skeletal tissues under different disease conditions.^[20]^ A biochemical analysis conducted by Pathak et al on the closure zone of cranial sutures in 9 patients with craniosynostosis showed consistent increases in calcium, phosphorus, phospholipids, and chondroitin sulfate-A levels within the bone tissue of the affected cranial suture closure zone.^[21]^ Tzvetkov et al classified and identified 80 and 1 lipid ions with varying abundances in the joint tissues of juvenile and adult mice, respectively. It was found that phospholipase-activated platelet-activating factor (PAF) and its related metabolites were rich in the bone marrow in both age groups, while the lipid content in cortical bone was relatively low. Notably, lysolecithin was notably enriched in the growth plate. Studies have demonstrated that phospholipase 1 (PHOSPHO1) plays a vital role in generating the phosphate required for bone mineralization. Changes in the levels of various lipid ion derivatives, such as PC, SM, and hemolytic phosphatidylethanolamine, were observed to varying degrees. Furthermore, it was observed that hemolytic phospholipids containing linoleic acid and linolenic acid were particularly prominent in the bone marrow. These findings highlight the importance of phospholipid membrane composition not only in structural functionality but also in bone development metabolism and regulation of signal transduction. Furthermore, we acknowledge that our single-cell RNA sequencing analysis utilized physiological (normal) murine sutures to map the baseline osteogenic trajectory. While this approach establishes a critical reference for the homeostatic mechanisms – specifically the lipid-dependent metabolic state of the C4 subpopulation – it may not fully capture the pathological transcriptomic landscape of craniosynostosis. To validate the disease specificity of our findings, future studies should employ craniosynostosis mouse models, such as Twist1± or Fgfr2 mutants, to determine whether the depletion of the C4 population or disruption of the lipid-MORN5 axis acts as a definitive trigger for premature fusion. Despite this limitation, the core osteogenic signaling pathways (e.g., BMP and TGF-β) are highly conserved between mice and humans, supporting the translatability of these metabolic insights to human pathology.

As FDA-approved drugs, Statins are mainly prescribed for patients with abnormal cholesterol metabolism. Currently, research on its involvement in lipid metabolism is still ongoing. Previous studies have suggested that statin use can cause changes in cell membrane phospholipids, affecting cholesterol transportation and synthesis.^[22]^ In the study of craniosynostosis etiology, mutations in genes related to bone development are considered, with limited research on lipid-related metabolic abnormalities. Metabolic alterations have been found in various skeletal diseases,^[23]^ including osteoporosis, highlighting the increasing recognition of the connection between lipid metabolism and bone health. Therefore, it is crucial to regulate cellular and systemic lipid levels to maintain optimal bone homeostasis.

In conclusion, the formation and development of cranial sutures are complex processes. Phospholipids play a crucial role in the formation and maintenance of cranial sutures. Further research is necessary to understand the impact of gene mutations related to phospholipid metabolism and the dysregulation of associated pathways in craniosynostosis and related syndromes, enabling a thorough comprehension of the involvement of phospholipids in these disorders. Interpretation of drug-target analysis: our revised MR analysis targeting HMGCR showed a biologically plausible effect size (OR = 0.79) but lacked statistical significance (*P* > .05). This is a known limitation when applying MR to rare diseases with small GWAS sample sizes, which often results in insufficient power to detect moderate drug effects. However, the protective direction (OR < 1) aligns with our findings on VLDL phospholipids. Therefore, we interpret the statin result as exploratory evidence. To bridge the gap between this genetic signal and clinical potential, we employed network pharmacology (Figs. 9–15) to map the potential multi-target mechanisms of Rosuvastatin beyond simple lipid lowering. Our pharmacological predictions, particularly the strong binding affinity of Rosuvastatin to MORN5 and MAPK1 (<−5 kcal/mol), suggest a theoretical basis for drug repurposing. However, we acknowledge that these findings are currently associative and computational. To validate these in silico predictions, future experimental studies are essential. Specifically, we propose an experimental model using primary cranial mesenchymal stem cells derived from craniosynostosis murine models. These cells should be treated with varying concentrations of Rosuvastatin to assess: changes in osteogenic marker expression (e.g., Runx2, Alp); alterations in membrane lipid composition via lipidomics; and the rescue of premature fusion phenotypes. Until such wet-lab validation is conducted, Rosuvastatin should be viewed as a candidate for investigation rather than an immediate therapeutic strategy. Deciphering the regulation of these mechanisms can enhance our understanding of the temporal and spatial changes in the expression of craniosynostosis-related genes and guide the development of therapeutic strategies.

## Author contributions

**Conceptualization:** Yuqi Zhang.

**Data curation:** Zhimin Huang.

**Formal analysis:** Kaisai Tuerxun.

**Methodology:** Junhua Wang.

**Project administration:** Yuqi Zhang.

**Writing – original draft:** Kaisai Tuerxun.

## Supplementary Material


