# Supplementary material for: Association between craniosynostosis and phospholipid metabolism: Insights from single-cell and transcriptomic analysis
Source: Medicine (Baltimore). 2026 Mar 27;105(13):e48030. doi: 10.1097/MD.0000000000048030 (PMC13034944; doi:10.1097/MD.0000000000048030)
Supplement: Supplementary file 1 [file medi-105-e48030-s001.doc]

| **SNP** | **Chr** | **Position (BP)** | **Effect Allele** | **Other Allele** | **Beta (LDL)** | **SE** | **P-value** | **F-statistic** |
| --- | --- | --- | --- | --- | --- | --- | --- | --- |
| rs10045497 | 5 | 74636484 | A | C | 0.077 | 0.005 | 2.32E-44 | 208.9 |
| rs76475757 | 5 | 74637213 | C | T | 0.056 | 0.01 | 4.88E-07 | 30.3 |
| rs10515198 | 5 | 74641560 | A | G | 0.06 | 0.006 | 5.99E-22 | 96.4 |
| rs2303152 | 5 | 74641707 | A | G | 0.042 | 0.006 | 1.04E-09 | 43.7 |
| rs17244848 | 5 | 74643670 | C | T | 0.054 | 0.008 | 1.07E-09 | 41.9 |
| rs10474434 | 5 | 74644681 | T | G | 0.063 | 0.006 | 4.11E-22 | 105 |
| rs6453131 | 5 | 74644706 | G | T | 0.068 | 0.005 | 4.46E-35 | 172 |
| rs11742194 | 5 | 74646878 | T | C | 0.059 | 0.006 | 8.13E-22 | 98 |
| rs17238484 | 5 | 74648496 | T | G | 0.063 | 0.006 | 1.35E-21 | 102.3 |
| rs3846662 | 5 | 74651084 | G | A | 0.069 | 0.004 | 2.24E-69 | 331.6 |
| rs3846663 | 5 | 74655726 | T | C | 0.072 | 0.004 | 1.13E-75 | 380.8 |
| rs5909 | 5 | 74656175 | A | G | 0.062 | 0.009 | 4.93E-13 | 49.2 |
| rs12916 | 5 | 74656539 | C | T | 0.073 | 0.004 | 7.79E-78 | 372.1 |
| rs10474435 | 5 | 74657280 | C | T | 0.054 | 0.015 | 2.36E-03 | 12.9 |
